# The Accuracy and Capability of Artificial Intelligence Solutions in Health Care Examinations and Certificates: Systematic Review and Meta-Analysis

**DOI:** 10.2196/56532

**Published:** 2024-11-05

**Authors:** William J Waldock, Joe Zhang, Ahmad Guni, Ahmad Nabeel, Ara Darzi, Hutan Ashrafian

**Affiliations:** 1 Imperial College London London United Kingdom; 2 Institute of Global Health Innovation Imperial College London London United Kingdom

**Keywords:** large language model, LLM, artificial intelligence, AI, health care exam, narrative medical response, health care examination, clinical commissioning, health services, safety

## Abstract

**Background:**

Large language models (LLMs) have dominated public interest due to their apparent capability to accurately replicate learned knowledge in narrative text. However, there is a lack of clarity about the accuracy and capability standards of LLMs in health care examinations.

**Objective:**

We conducted a systematic review of LLM accuracy, as tested under health care examination conditions, as compared to known human performance standards.

**Methods:**

We quantified the accuracy of LLMs in responding to health care examination questions and evaluated the consistency and quality of study reporting. The search included all papers up until September 10, 2023, with all LLMs published in English journals that report clear LLM accuracy standards. The exclusion criteria were as follows: the assessment was not a health care exam, there was no LLM, there was no evaluation of comparable success accuracy, and the literature was not original research.The literature search included the following Medical Subject Headings (MeSH) terms used in all possible combinations: “artificial intelligence,” “ChatGPT,” “GPT,” “LLM,” “large language model,” “machine learning,” “neural network,” “Generative Pre-trained Transformer,” “Generative Transformer,” “Generative Language Model,” “Generative Model,” “medical exam,” “healthcare exam,” and “clinical exam.” Sensitivity, accuracy, and precision data were extracted, including relevant CIs.

**Results:**

The search identified 1673 relevant citations. After removing duplicate results, 1268 (75.8%) papers were screened for titles and abstracts, and 32 (2.5%) studies were included for full-text review. Our meta-analysis suggested that LLMs are able to perform with an overall medical examination accuracy of 0.61 (CI 0.58-0.64) and a United States Medical Licensing Examination (USMLE) accuracy of 0.51 (CI 0.46-0.56), while Chat Generative Pretrained Transformer (ChatGPT) can perform with an overall medical examination accuracy of 0.64 (CI 0.6-0.67).

**Conclusions:**

LLMs offer promise to remediate health care demand and staffing challenges by providing accurate and efficient context-specific information to critical decision makers. For policy and deployment decisions about LLMs to advance health care, we proposed a new framework called RUBRICC (Regulatory, Usability, Bias, Reliability [Evidence and Safety], Interoperability, Cost, and Codesign–Patient and Public Involvement and Engagement [PPIE]). This presents a valuable opportunity to direct the clinical commissioning of new LLM capabilities into health services, while respecting patient safety considerations.

**Trial Registration:**

OSF Registries osf.io/xqzkw; https://osf.io/xqzkw

## Introduction

The advent of large language models (LLMs), such as Chat Generative Pretrained Transformer (ChatGPT; OpenAI), has generated extraordinary interest worldwide and transformed the landscape of artificial intelligence (AI). This foremost positioning of transformer models in the public and academic consciousness has been achieved by the remarkable ability of generative artificial intelligence (genAI) models to create new content with human-like semantics and syntax, alongside the capability to accurately replicate learned knowledge in narrative text. Numerous applications in medical research [1], medical education [1], clinical communication or consultation [2], and even diagnosis and risk prediction tasks [2] have been demonstrated to date. There is great positive potential for genAI across all of these pathways and great promise to relieve the increasing pressures and shortage of clinical expertise in health care systems worldwide [2].

The ability of genAI to answer medical examination questions is of particular interest. First, such examinations serve as the gateway for professional qualification. Written examination questions replicate complex clinical scenarios in narrative form and may include the possibility of multiple reasonable differential diagnoses (multiple choice) or require ranking of medically appropriate responses (single-best answer) according to not just clinical knowledge but also contextual decision-making and medical ethics. For decades, this type of examination has been the ultimate test of human clinical judgment and depth of knowledge. The performance of LLMs in this context has far-reaching implications for how medical education is delivered. Second, these expert-developed and expert-validated question-answer pairs are a coherent substitute for real-world training data written in narrative form and may serve to tune genAI models with a clinical consultation, communication, or diagnostic function. This is exemplified by Google’s use of medical examination questions to train and test Medical Patient Language Model 2 (Med-PaLM 2) [3]. Finally, these same validated questions are a ready-made benchmark for assessing LLM capabilities in future clinical or medical education–related tasks.

However, the use of LLMs is not without risk. They have a propensity to “hallucinate” false information and produce potentially dangerous inaccuracies [4]. In addition, LLMs are created through a process of pretraining on vast existing text corpuses to enable a general understanding of syntax and semantics. Although models may undergo fine-tuning for particular tasks or domains, this process does not modify the underlying “learned” knowledge but adjusts weights to adapt the model’s outputs for a required context. As such, the underlying embedding of our current societal state means that models will also encode societal biases, which will certainly include biases seen in health care provision and outcomes [5]. An understanding of how these problems manifest in real-world tasks is key to developing mitigations and to establish risks and benefits of the use of LLMs in different medical areas.

We conducted a systematic review of LLM accuracy, as tested under health care examination conditions, as compared to known human performance standards. We assessed the reporting quality and risk of bias within existing studies and synthesized a discussion of pitfalls and performance concerns, as reported by study investigators. We discussed how the observed LLM performance impacts medical education and genAI-enabled clinical consultation and recommended a framework for the conduct of future research in this area. In response to this rapidly progressing field, we aimed to establish a baseline performance and quality standard for the current generation of LLMs in narrative medical response tasks.

## Methods

### Study Design

The systematic review was conducted according to a registered protocol and was reported according to the PRISMA (Preferred Reporting Items for Systematic Reviews and Meta-Analyses) statement [6]. The protocol was registered with the Open Science Framework (OSF) [7], under the title “How Accurate are Artificial Intelligence LLMs When Applied to Healthcare Exams and Certificates?”, with the secondary research questions “What is the performance of LLM in comparison to required examination standards for humans?” and “What are the primary discovered weaknesses of LLM in addressing narrative health care examination scenarios that may be pertinent to real-world performance in clinical scenarios?”

### Eligibility Criteria

The inclusion criteria were all papers up until September 10, 2023, published in English language journals that describe the use of AI solutions in health care examinations and certificates. As reflected in the Medical Subject Headings (MeSH) terms used, the authors screened the manuscripts for “artificial intelligence,” which could be described in the following possible ways: “ChatGPT,” “GPT,” “LLM,” “large language model,” “machine learning,” “neural network,” “Generative Pretrained Transformer,” “Generative Transformer,” “Generative Language Model,” or “Generative Model.” The exclusion criteria were as follows: the assessment was not a health care examination, there was no LLM, there was no evaluation of comparable success accuracy, and the literature was not original research (ie, commentary, editorials, reviews). We assessed LLMs, first, as applied to health care examinations and, by extension, as applied to clinical problems, including those encountered by individual patients and clinicians, and the likely impact on future medical education. We assessed the outcome of the accuracy of examination response performance and an intervention of the use of LLMs to answer narrative health care examination questions. The additional variable(s)/covariate(s) to consider were the name and country of medical examination; the “pass mark” and other score boundaries for each medical examination; the average and intervals of human performance for each medical examination that included benchmarks; the identity of LLMs; LLM characteristics, including parameter size; and any fine-tuning for the LLMs prior to testing.

### Information Sources

The search included all papers up until September 10, 2023, at which point a preliminary search was conducted and piloting of the study selection process was commenced using MEDLINE/PubMed, CINAHL, ClinicalTrials.gov, Embase, and Google Scholar.

### Search Strategy

The literature search included the following MeSH terms used in all possible combinations: “artificial intelligence,” “ChatGPT,” “GPT,” “LLM,” “large language model,” “machine learning”, “neural network”, “Generative Pre-trained Transformer”, “Generative Transformer,” “Generative Language Model,” “Generative Model,” “medical exam,” “healthcare exam,” and “clinical exam.” Two authors (WJW and AG) independently identified relevant studies, and any discrepancies were resolved by consensus with the help of a third author (HA)

### Selection Process

Screening reliability and duplicate removal were maintained by 2 independent screeners reviewing abstracts (WJW and AG), with divergent screener decisions reconciled by a third master screener (HA). Abstracts were downloaded and screened in Covidence software [8] using .rsi and .csv files. Two independent authors (WJW and AG) performed full-text manuscript screening following abstract screening, with discrepancies resolved by consultation with the lead author (HA).

### Data Collection, Data Items, and Data Synthesis

Two reviewers (WJW and AG) extracted and synthesized comparative accuracy data from the reviews on Covidence. No automation tools were used. The 2 authors independently extracted data from relevant studies, and any discrepancies were resolved by consensus with the help of a third author (HA) Sensitivity, accuracy, and precision data were extracted, including relevant CIs. The meta-analysis pooling of aggregate data used the random-effects inverse-variance model with DerSimonian-Laird estimate of tau^2^. The software used to conduct the meta-analysis was Stata Statistical Software Release 15 (StataCorp).

### Risk-of-Bias Assessment and Reporting Bias Assessment

The QUADAS-2 tool [9] was used for the systematic evaluation and assessment of the risk of bias and concerns regarding answer accuracy for clinical examination questions. The evaluation enabled adjudication of the applicability and bias concerns regarding reference standards and training data selection. Two independent authors performed the risk-of-bias assessment, with discrepancies resolved by consultation with the lead author (HA).Results

## Results

### Study Screening

**Figure 1:**
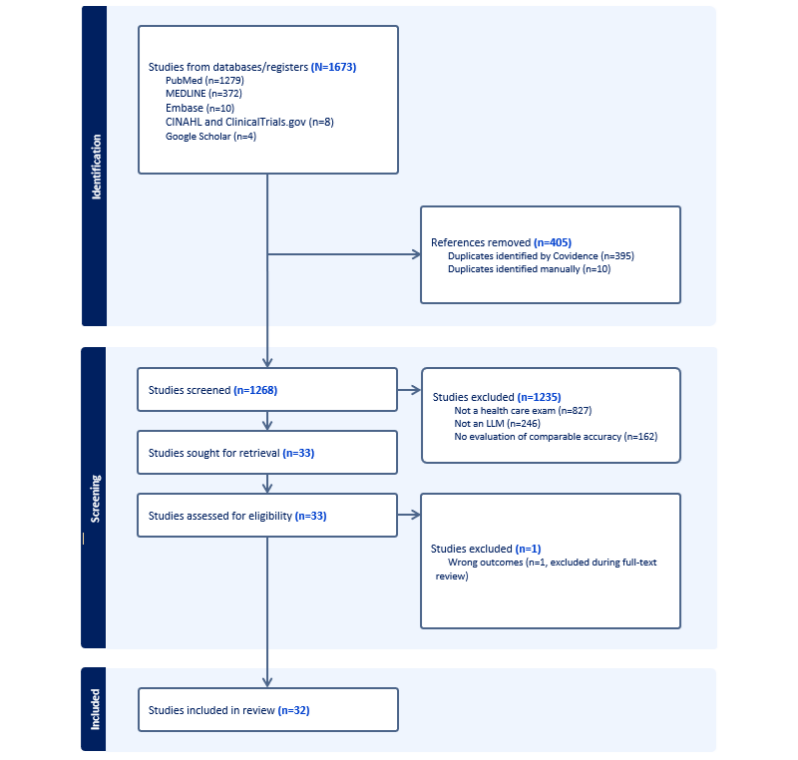
Study selection based on PRISMA guidelines. PRISMA: Preferred Reporting Items for Systematic Reviews and Meta-Analyses.

Based on PRISMA guidelines, the search identified 1673 relevant citations. After removing duplicate results, 1268 (75.8%) papers were screened for titles and abstracts, and 32 (2.5%) [3,10-40] studies were included for full-text review (see Figure 1 and Table S1 in Multimedia Appendix 1).

The LLMs represented in this systematic literature review were Flan-PaLM 2 [3], Generative Pretrained Transformer (GPT)-Neo [10], ChatGPT [11-35], Google Bard [13], Bing Chat [13], PubMedGPT (Stanford University) [36], BioLinkBERT [37] (BERT stands for Bidirectional Encoder Representations from Transformers), PubMedBERT [38], Galactica [39], and DRAGON (Deep Bidirectional Language-Knowledge Graph Pretraining) [40]. All these models are commercial, except BioLinkBERT, GPT-Neo, and DRAGON. The majority of LLMs used in medical examination tasks were pretrained, closed source models, developed and released by commercial organizations, such as ChatGPT. There was no prompt engineering described by the majority of the studies [11,13,15-35] when using ChatGPT, but Kung et al [12] and Gilson et al [14] specifically introduced prompt engineering to mitigate concerns about model “hallucinations” [41]. Stanford University’s PubMedGPT 2.7B [36] is an LLM trained on PubMed abstracts and Pile. Flan-PaLM 2 [3], PubMedGPT [36], DRAGON [40], BioLinkBERT [37], Galactica [39], PubMedBERT [38], and GPT-Neo [10] were all evaluated using the same 12,723 United States Medical Licensing Examination (USMLE) open source question dataset [42]. BioLinkBERT [37] is a self-supervised pretraining bidirectional system that leverages graph structures in PubMed. PubMedBERT [38] is a BERT-style model trained on PubMed, while Galactica [39] is a GPT-style model trained on scientific literature that is 44 times the size of PubMedGPT 2.7B [36].

### Precision, Sensitivity, and Accuracy

#### Precision

When assessing the precision of LLMs in all examinations, 2 (6.3%) studies had an overall precision of 0.61 (CI 0.55-0.67) across 189 questions, with a tau^2^ heterogeneity of 0.0018 and an *I*^2^ variation attributable to a heterogeneity of 99.6%.

#### Sensitivity

When assessing the sensitivity of LLMs in all examinations, 2 (6.3%) studies had an overall sensitivity of 1.00 (CI 1.00-1.00) across 189 questions, with a tau^2^ heterogeneity of 0.0000 and an *I*^2^ variation attributable to a heterogeneity of 0%.

#### Accuracy

**Figure 2:**
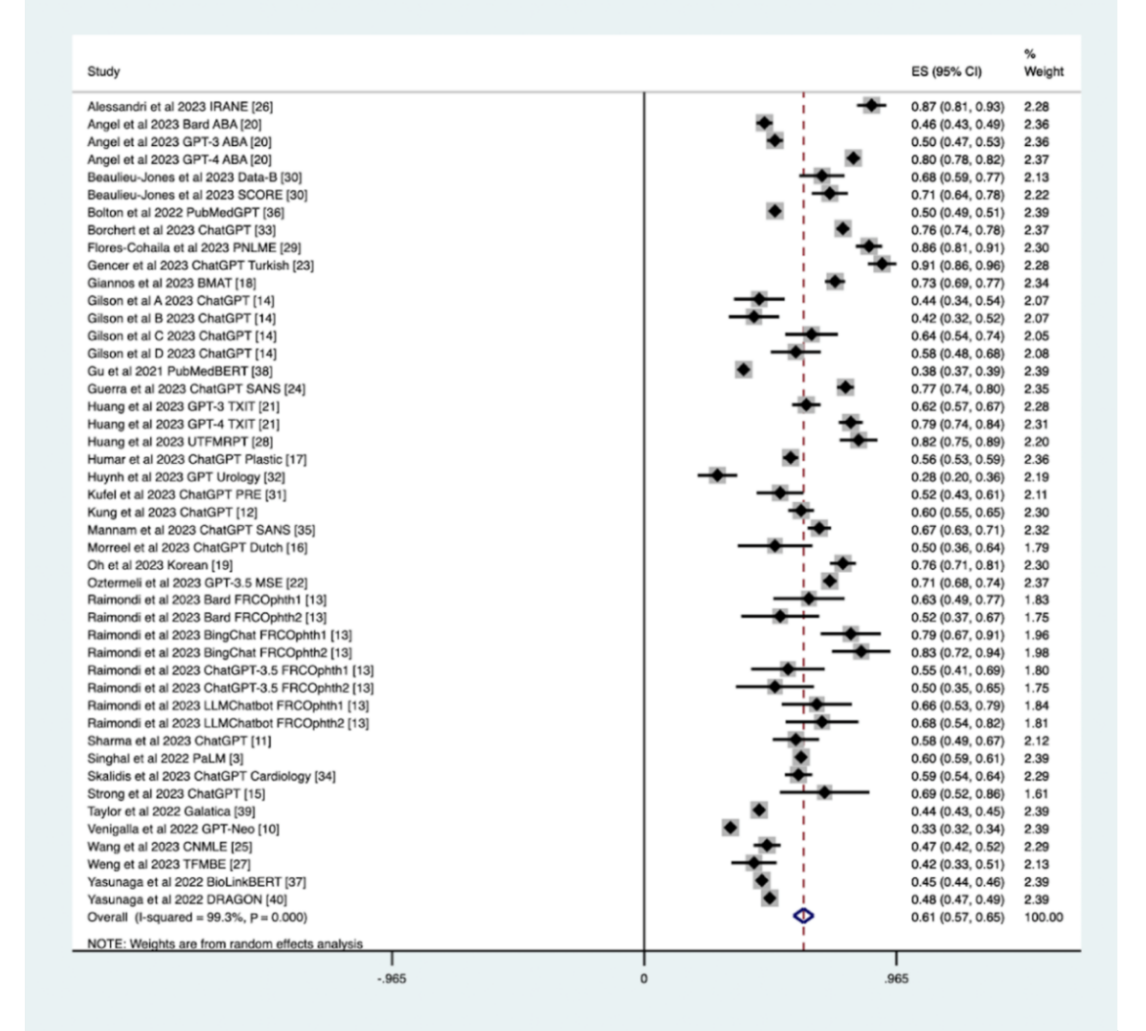
Forest plot of the accuracy of LLM performance on all medical examinations. ABA: American Board of Anesthesiology; BERT: Bidirectional Encoder Representations from Transformers; ChatGPT: Chat Generative Pretrained Transformer; CNMLE: Chinese National Medical Licensing Examination; DRAGON: Deep Bidirectional Language-Knowledge Graph Pretraining; FRCOphth: Fellowship of the Royal College of Physicians and Surgeons (Ophthalmology); GPT: Generative Pretrained Transformer; IRANE: Italian Residency Admission National Exam; LLM: large language model; MSE: medical specialty examination; PaLM: Patient Language Model 2; PNLME: Peruvian National Licensing Medical Examination; PRE: Polish radiology examination; SANS: Self-Assessment Neurosurgery; SCORE: Surgical Council on Resident Education; TFMBE: Taiwan Family Medicine Board Exam; TXIT: Radiation Oncology in-Training; UTFMRPT: University of Toronto Family Medicine Residency Progress Test.

The overall LLM examination performance, USMLE accuracy, and ChatGPT accuracy were all evaluated by substudy meta-analysis, with question counts moderated for double-counting across multiple substudies. When assessing the accuracy of LLMs in all examinations, 47 substudies had an overall accuracy of 0.61 (CI 0.58-0.64) across 22,347 questions, with a tau^2^ heterogeneity of 0.0088 and an *I*^2^ variation attributable to a heterogeneity of 100% (Table 1 and Figure 2).

**Table 1:** LLM^a^ meta-analysis substudies.

Study and substudies		Questions, n	Accuracy
Alessandri Bonetti et al [26]; IRANE (Italian Residency Admission National Exam)		140	0.87
**Angel et al [20]**			
	Bard American Board of Anesthesiology (ABA)	1000	0.46
	GPT^b^-3 ABA	1000	0.50
	GPT-4 ABA	1000	0.80
**Beaulieu-Jones et al [30]**			
	Data-B	112	0.68
	SCORE (Surgical Council on Resident Education)	167	0.71
**Bolton et al [36]**			
	PubMedGPT	12,723	0.50
	ChatGPT^c^	1217	0.76
Flores-Cohaila et al [29]; Peruvian National Licensing Medical Examination (PNLME)		180	0.86
Gencer et al [23]; Turkish ChatGPT		105	0.91
Giannos et al [18]; BioMedical Admissions Test (BMAT)		509	0.73
**Gilson et al [14]**			
	ChatGPT A	100	0.44
	ChatGPT B	100	0.42
	ChatGPT C	87	0.64
	ChatGPT D	102	0.58
Gu et al [38]; PubMedBERT^d^		12,723	0.38
Guerra et al [24]; ChatGPT Self-Assessment Neurosurgery (SANS)		643	0.77
**Huang et al [21]**			
	GPT-3 Radiation Oncology in-Training (TXIT)	300	0.62
	GPT-4 TXIT	300	0.79
Huang et al [28]; University of Toronto Family Medicine Residency Progress Test (UTFMRPT)		108	0.82
Humar et al [17]; ChatGPT plastic surgery		1129	0.56
Huynh et al [32]; GPT urology		135	0.28
Kufel et al [31]; ChatGPT Polish radiology examination		120	0.52
Kung et al [12]; ChatGPT		376	0.60
Mannam et al [35]; ChatGPT SANS		427	0.67
Morreel et al [16]; ChatGPT Dutch		47	0.50
Oh et al [19]; ChatGPT Korean		280	0.76
Oztermeli et al [22]; GPT-3.5 medical specialty examination (MSE)		1177	0.71
**Raimondi et al [13]**			
	Bard Fellowship of the Royal College of Physicians and Surgeons (Ophthalmology), or FRCOphth, part 1	48	0.63
	Bard FRCOphth part 2	43	0.52
	Bing Chat FRCOphth part 1	48	0.79
	Bing Chat FRCOphth part 2	43	0.83
	ChatGPT-3.5 FRCOphth part 1	48	0.55
	ChatGPT-3.5 FRCOphth part 2	43	0.50
	LLM chatbot FRCOphth part 1	48	0.66
	LLM chatbot FRCOphth part 2	43	0.68
Sharma et al [11]; ChatGPT		119	0.58
Singhal et al [3]; Med-PaLM 2^e^		12,723	0.60
Skalidid et al [34]; ChatGPT cardiology		340	0.59
Strong et al [15]; ChatGPT		28	0.69
Taylor et al [39]; Galactica		12,723	0.44
Venigalla et al [10]; GPT-Neo		12,723	0.33
Wang et al [25]; Chinese National Medical Licensing Examination (CNMLE)		360	0.47
Weng et al [27]; Taiwan Family Medicine Board Exam (TFMBE)		125	0.42
Yasunaga et al [37]; BioLinkBERT		12,723	0.45
Yasunaga et al [40]; DRAGON^f^		12,723	0.48

^a^LLM: large language model.

^b^GPT: Generative Pretrained Transformer.

^c^ChatGPT: Chat Generative Pretrained Transformer.

^d^BERT: Bidirectional Encoder Representations from Transformers.

^e^Med-PaLM 2: Medical Patient Language Model 2.

^f^DRAGON: Deep Bidirectional Language-Knowledge Graph Pretraining.

#### USMLE Accuracy

When assessing the accuracy of LLMs in the USMLE, 14 substudies had an overall accuracy of 0.51 (CI 0.46-0.56) across 13,535 questions, with a tau^2^ heterogeneity of 0.0080 and an *I*^2^ variation attributable to a heterogeneity of 100%.

#### ChatGPT Accuracy

When assessing the accuracy of ChatGPT on medical examinations, 32 substudies had an overall accuracy of 0.64 (CI 0.6-0.67) across 9824 questions, with a tau^2^ heterogeneity of 0.0128 and an *I*^2^ variation attributable to a heterogeneity of 100%.

#### Bias and Narrative Reporting

**Figure 3:**
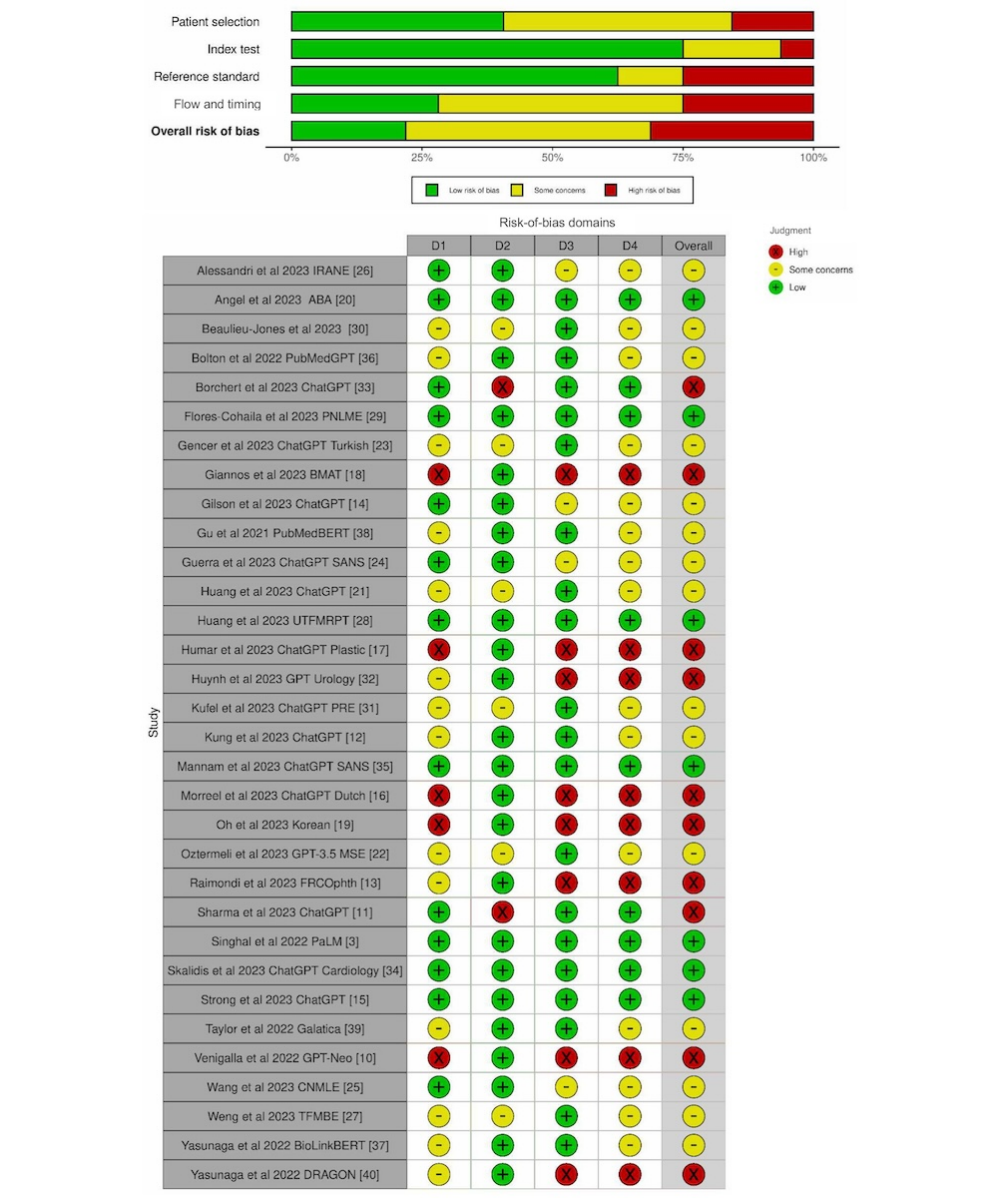
Risk-of-bias. ABA: American Board of Anesthesiology; BERT: Bidirectional Encoder Representations from Transformers; ChatGPT: Chat Generative Pretrained Transformer; CNMLE: Chinese National Medical Licensing Examination; DRAGON: Deep Bidirectional Language-Knowledge Graph Pretraining; FRCOphth: Fellowship of the Royal College of Physicians and Surgeons (Ophthalmology); GPT: Generative Pretrained Transformer; IRANE: Italian Residency Admission National Exam; LLM: large language model; MSE: medical specialty examination; PaLM: Patient Language Model 2; PNLME: Peruvian National Licensing Medical Examination; PRE: Polish radiology examination; SANS: Self-Assessment Neurosurgery; SCORE: Surgical Council on Resident Education; TFMBE: Taiwan Family Medicine Board Exam; TFMR: Toronto Family Medicine Residency; TXIT: Radiation Oncology in-Training.

Among the 32 studies that underwent QUADAS-2 [9,43] risk-of-bias evaluation (Figure 3), only 11 (24.4%) were eligible for meta-analysis. Overall, 10 (31.3%) studies were found to have high bias, 15 (46.9%) studies were found to have some concerns of bias, and 7 (21.9%) studies were found to have low bias. In addition, 3 (9.4%) studies referred to concerns about “hallucinations,” but none described the effect nor referred to softer themes, such as empathy. No studies evaluated bias systematically. None of the reviewed literature was systematic reviews, so a TRIPOD (Transparent Reporting of a Multivariable Prediction Model for Individual Prognosis or Diagnosis) adherence to reporting standards analysis [44] was not conducted.

## Discussion

### Principal Findings

Our meta-analysis suggests that LLMs are able to perform with an overall medical examination accuracy of 0.61 (CI 0.58-0.64) and a USMLE accuracy of 0.51 (CI 0.46-0.56), while ChatGPT can perform with an overall medical examination accuracy of 0.64 (CI 0.6-0.67). We quantified the accuracy of LLMs in responding to health care examination questions and evaluated the consistency and quality of study reporting. The majority of LLMs used in medical examination tasks were pretrained, closed source models, developed and released by commercial organizations, such as ChatGPT. However, we found that minimal research has explored bias, “hallucination,” and holistic evaluation of the LLMs themselves. Moreover, neither the risk of bias nor holistic evaluation frameworks exist for LLMs themselves.

There are inherent challenges to integrating LLMs into the education and clinical decision support of human doctors. Use cases for LLMs include grading, detection, prediction, and content generation [45], but the application of these capabilities to the sociocultural elements of medicine are complex. Doctors offer empathetic relationships and formulate clinical reasoning in a more transparent way than current LLMs, raising concerns that the introduction of LLMs will undermine doctor-patient rapport [46] and trust in the ethical compliance of the health care system. LLMs can automate the generation of text content, which offers opportunities to enhance student answer marking and provide responsive learning assistant chat features [45]. However, these features lack transparency, prompting distrust in decision-making [47], and a lack of evidence generation around student engagement [48]. Although these training and infrastructure hurdles must be overcome, there is immense potential for personalized learning experiences with augmented and virtual reality, alongside enhanced curriculum implementation [49].

Medical examinations are not the same as medical practice [50]. The tests that are designed to confirm a human’s suitability to practice medicine independently may not be appropriate for an LLM; real-world practice involves greater pathophysiological complexity, diverse holistic care considerations, and important ethical accountability frameworks to ensure empathetic patient-centered health services. Here, we demonstrated LLM capabilities in question-and-answer tasks according to established international benchmarks. Single-best answer questions are designed to simulate clinical decision-making, but there is a lack of relevance of examination questions to real-world tasks [5]. Current models are trained on an unregulated range of both narrow and broad data sets to perform tasks with translational evidence, which currently have unclear significance in clinical practice [5]. LLMs are not yet ready to be a proxy for human education, as questions simplify and isolate scenarios in an imperfect representation of real situations encountered by clinicians. However, the success of LLMs may justify a reconfiguration or even a disruption of medical training. This might involve an initial move toward formative assessments in view of the limitations of summative assessments exposed by the success of LLMs in the USMLE [3]; rather, when offered access to a hitherto untapped wealth of medical information, the role of the doctor may be able to provide judicious medical decisions when presented with intelligent and superintelligent LLM-generated treatment strategies.

Virtual and remote learning opportunities will be enhanced by LLMs [49], but bias, cost, and “hallucination” are the major obstacles to their application in health care. The definition of the threshold for acceptable clinical deployment varies across clinical scenarios and disease states due to the variation in the acceptable tolerance of error. LLMs are developed with parameters that reflect the established sociocultural inequalities in our society and can be perpetuated in LLMs without further intervention. Solutions such as LLM-focused data governance strategies within current and future guidelines and novel approaches, including the use of synthetic data, will likely be needed to ensure those underserved by current data collection pools are not discriminated against in the behavior of the LLMs [51]. With estimates suggesting that US $5 million of graphical processing units (GPUs) [52] are needed at minimum for 1 LLM, their impressive capabilities are unlikely to be ubiquitous across health systems, such as the UK National Health Service (NHS), and may exacerbate inequalities. Finally, there is an inherent danger of “hallucination” with LLMs, undermining the protection of patient data and accurate contributions to live clinical scenarios [53].

### Study Limitations

The studies failed to explore the main barriers to LLM implementation in clinical practice, including bias, “hallucinations,” usability, cost, and privacy. The extensive variation between studies in the terminology, methodology, outcome measures, and data interpretability could be explained by a lack of consensus on how to conduct and report LLM studies. We have concerns over the reliability of these studies and the small volume of eligible studies for comparison. The lack of consistency in accuracy reporting between studies obstructed evaluation of the relative strengths of each method. There is an inherent challenge in evaluating technology with substantial commercial potential due to producers’ understandable reluctance about publishing sensitive details that may enable reproducibility but undermine commercial advantage. Our review concentrated on health care examination LLM performance and so did not account for LLM capability in more generalist evaluations that may still have valuable insights for optimizing health care capabilities.

### Future Work

For policy and deployment decisions of LLMs to advance health care, we propose a new framework called *RUBRICC* (Regulatory, Usability, Bias, Reliability [Evidence and Safety], Interoperability, Cost, Codesign–Patient and Public Involvement and Engagement [PPIE]). See Multimedia Appendix 2.

#### Regulatory

LLMs have unique evaluation requirements. Medicines and Healthcare products Regulatory Agency (MHRA) device standards may categorize some clinical LLMs as type 2b devices [54], although medical knowledge progression (eg, National Institute for Health and Care Excellence [NICE] guidelines) may require the recall of LLMs due to their capabilities being contained by period updates. Moreover, specific LLM standards for clinical commissioning are yet to be defined. It is important to forecast probable applications of LLMs, such as medical chatbots, clinical documentation, obtaining insurance preauthorization [55], and reviewing research papers [56]. Therefore, the regulatory responsibilities to patient safety and privacy will demand scrutiny on the grounds of LLMs’ complexity, hardware, privacy, and real-time adaptation [55]. Developing rigorous and robust regulatory standards will require the commitment and input of key stakeholders, including clinicians, engineers, researchers, ethicists, health policymakers, and patients. Importantly, standards must be regularly adapted and revised to meet the rapidly advancing and evolving nature of LLMs.

#### Usability

Early adopter contexts will also likely be when the LLM is a clinical decision support tool integrated into various clinical contexts ranging from triage and differential diagnoses to imaging and medication decisions. Different geographies may apply these technologies differently, from the United States’ insurance-based federated health landscape, which will likely apply LLMs to local health systems, in contrast to national data connectivity, which offers en masse precision LLM use across specialties, systems, and care tiers, such as in Estonia or the United Kingdom’s NHS [57]. Academia will also be impacted, with publication assistance accelerating the role of LLM-coauthored literature [56].

#### Bias

The systematic review literature deals in terms of bias, which represents the content and function of an AI. The bias discussions in the included papers focused on the following variables: *within-item anchoring bias*, *grounding bias*, *chain-of-thought bias*, and *demographic bias*. By contrast, risk characterizes the contextual impact of an LLM in conversations that inform commissioning of generative medical AI and aligns with current regulatory frameworks for current and future AI tools [56]. Singhal et al [3] evaluated Med-PaLM 2 using the following LLM answer risk framework: *more inaccurate/irrelevant information*, *omits more information*, *more evidence of demographic bias*, *greater extent of harm*, *greater likelihood of harm.* A key consideration is the risk matrix of LLM errors. There are unique requirements for LLM reporting that do not easily map onto established criteria, such as the Standards for Reporting of Diagnostic Accuracy Study (STARD) 2015 checklist [58], and can be incorporated into the upcoming STARD-AI [59]. Associated challenges related to bias include “hallucination” and privacy, threatening the reliability of these LLM services.

#### Reliability

##### Evidence

Differences in reference standards and thresholds for diagnostic accuracy make comparison of LLM studies difficult in this nascent field, undermining the pathway to integration into health systems. These problems can only be addressed by specific reporting standards for AI studies [59,60], with design accuracy to address issues of reproducibility, transparency, and efficacy [61]. Further evidence is needed to develop reliable guidelines [62]. We therefore await guidelines that accommodate LLM utility to enable higher-quality and more consistent reporting, which in turn will empower the MHRA and the Food and Drug Administration (FDA) to be able to evaluate LLM risk. Specifically, the development of AI-specific risk-of-bias tools, such as QUADAS-AI, will aid in establishing the risk of bias for evidence synthesis of clinical LLM studies, allowing clinically relevant conclusions to be drawn more confidently [43].

##### Safety

Multidisciplinary secure data environments (SDEs) [63] must be established with cybersecurity standards to assuage recognized concerns about AI manipulation and displacement of human welfare priorities [64]. There remain established concerns about the regulated integration of LLMs into established clinical workstreams in view of “hallucination” concerns, which will require a quality management system to ensure compliance with best practices to mitigate risk to patients.

#### Interoperability

Although data flows in the NHS have been mapped [65], there is a growing demand for infrastructural transformation to reduce data inequalities and avoid the digital exclusion of unrepresented and underprivileged groups. A particular challenge includes multimodal data linkages and interoperability with integration of LLM tools in multiple different scenarios across the health service. One must be careful to consider how secondary or primary care data might be used differently to inform population health tools.

#### Cost

The economic considerations for LLMs can be organized into procurement, data processing, housing and cloud storage, management, and usability costs. Training costs have declined around 80% on models similar to ChatGPT-3 over the past 3 years [62]. The input cost is the number of tokens passed as prompts to the application programming interface (API), and the output cost is dependent on the number of tokens returned [63]. Therefore, for medical free-text record summarization, there is a large input cost dominated by the high quantity of tokens for each prompt. Self-hosted LLMs incur cloud service costs to run the models; it is notable that ChatGPT-4 (32 context length) is priced at US $60 input cost (per million tokens) and US $120 output cost (per million tokens) [66]. Further costs to consider include fine-tuning, which is most effective in improving performance on low-parameter models [67]; the clinical commissioning decisions related to these costs will be linked to the quality-adjusted life years (QALYs) associated with incremental performance improvements.

#### Codesign-PPIE

Public trust in LLMs can be built through a codesign process, adhering to INVOLVE [68] values, through respect, support, transparency, responsiveness, fairness of opportunity, and accountability. AI raises challenges for the codesign processes due to the disproportionate emphasis on procedures, patients lacking genuine understanding, and concerns AI may exacerbate inequalities; this is best resolved by a focus on sociotechnical values and design humility to acknowledge to patients what the proposed technology cannot achieve for them [69]. Meanwhile, doctor-patient rapport will likely be enhanced due to LLMs alleviating administrative tasks and helping clinicians answer patient questions [70]. RUBRICC is a nascent area of work that will undergo further development to enable utility and impact in the field.

### Conclusion

LLMs offer promise to remediate health care demand and staffing challenges by providing accurate and efficient context-specific information to critical decision makers. However, progress is obstructed by inconsistent reporting and an imbalance of resources between commercial interests and public sector regulators to independently evaluate potential LLM services. The ability of LLMs to pass the USMLE does not mean that the models answer useful questions to practicing clinicians [71]. Although initial results show impressive accuracy in isolated studies, there is an immediate need for a framework, such as RUBRICC, to evaluate this emergent technology and facilitate robust clinical commissioning decisions to benefit patients.

## Appendix

Multimedia Appendix 1 Summary of studies selected.

Multimedia Appendix 2 RUBRICC (Regulatory, Usability, Bias, Reliability [Evidence and Safety], Interoperability, Cost, Codesign–Patient and Public Involvement and Engagement [PPIE]), a framework for LLM clinical policy decisions. AI: artificial intelligence; LLM: large language model.

Multimedia Appendix 3 PRISMA (Preferred Reporting Items for Systematic Reviews and Meta-Analyses) checklist.

## Abbreviations

AI: artificial intelligence
BERT: Bidirectional Encoder Representations from Transformers
ChatGPT: Chat Generative Pretrained Transformer
DRAGON: Deep Bidirectional Language-Knowledge Graph Pretraining
genAI: generative artificial intelligence
GPT: Generative Pretrained Transformer
LLM: large language model
Med-PaLM 2: Medical Patient Language Model 2
MeSH: Medical Subject Headings
NHS: National Health Service
PRISMA: Preferred Reporting Items for Systematic Reviews and Meta-Analyses
RUBRICC: Regulatory, Usability, Bias, Reliability [Evidence and Safety], Interoperability, Cost, Codesign–Patient and Public Involvement and Engagement (PPIE)
STARD: Standards for Reporting of Diagnostic Accuracy Study
USMLE: United States Medical Licensing Examination

## Data Availability

The authors declare that all the data included in this study are available within the paper and multimedia appendices. Further information on research design is available in the Nature Research Reporting Summary linked to this paper.
