# Supplementary material for: The Accuracy and Capability of Artificial Intelligence Solutions in Health Care Examinations and Certificates: Systematic Review and Meta-Analysis
Source: J Med Internet Res. 2024 Nov 5;26:e56532. doi: 10.2196/56532 (PMC11576595; doi:10.2196/56532)
Supplement: Multimedia Appendix 1 [file jmir_v26i1e56532_app1.docx]

**Table S1.** Study details and accuracy.

| Study | LLM^a^ | Training method | Examination | Accuracy (%) |
| --- | --- | --- | --- | --- |
| Singhal et al [1] | Med-PaLM 2^b^ | We performed detailed human evaluations on long-form questions along multiple axes relevant to clinical applications. In pair-wise comparative ranking of 1066 consumer medical questions, physicians preferred Med-PaLM 2 answers to those produced by physicians on 8 of 9 axes pertaining to clinical utility (*P*<.001). | USMLE^c^ | 67.6 |
| Venigalla <et> al [2] | GPT^d^-Neo | An LLM of similar size and architecture as BioMedLM but trained on Pile and therefore not domain specific. | USMLE | 33.3 |
| Sharma et al [3] | ChatGPT | Considering the widespread use of ChatGPT and the reliance people place on it, this study determined how reliable ChatGPT can be for answering complex medical and clinical questions. USMLE questionnaires were used, obtained results using 2-way ANOVA and post hoc analysis. Both showed systematic covariation between format and prompt. | USMLE | 58.8 |
| Kung et al [4] | ChatGPT | 376 publicly available test questions from the June 2022 sample examination release, termed “USMLE-2022,” obtained from the official USMLE website. | USMLE | 60.0 |
| Raimondi et al [5] | ChatGPT-3.5, Google Bard, Bing Chat | Multiple-choice questions from the Royal College of Ophthalmologists website, covering both part 1 and part 2 examinations. | Fellowship of the Royal College of Physicians and Surgeons (Ophthalmology), or FRCOphth, part 1 and part 2 examinations | - LLM chatbots: 65.5 for part 1 and 67.6 for part 2 - ChatGPT-3.5: 55.1 for part 1 and 49.6 for part 2 - Google Bard: 62.6 for part 1 and 51.9 for part 2 - Bing Chat: 78.9 for part 1 and 82.9 for part 2 |
| Gilson et al [6] | ChatGPT | 4 data sets: AMBOSS-Step1, AMBOSS-Step2, National Board of Medical Examiners (NBME)-Free-Step1, and NBME-Free-Step2. | USMLE | - AMBOSS Step1: 44.0 - AMBOSS Step2: 42.0 - NBME Free Step1: 64.4 - NBME Free Step2: 57.8 |
| Strong et al [7] | ChatGPT | 14 multipart cases were selected from clinical reasoning examinations administered to preclerkship medical students between 2019 and 2022. | USMLE | 69.0 |
| Morreel et al [8] | ChatGPT | Multiple-choice examination for the course of family medicine in the 3rd bachelor year of the medical curriculum at Antwerp University. The examination consisted of 47 questions (Dutch language) with 4 possible answers and with a pass mark of 62.5%. | Dutch Family Medicine Exam | 50.0 |
| Humar et al [9] | ChatGPT | Plastic surgery in-service examinations from 2018 to 2022 were used as a question source. For each question, the stem and all multiple-choice options were imported into ChatGPT. | Plastic surgery in-service examinations from 2018 to 2022 | 55.8 |
| Giannos et al [10] | ChatGPT | Recent public resources (2019-2022) were used to compile a data set of 509 questions from the BioMedical Admissions Test (BMAT), the Test of Mathematics for University Admission (TMUA), the Law National Aptitude Test (LNAT), and the Thinking Skills Assessment (TSA), covering diverse topics in aptitude, scientific knowledge and applications, mathematical thinking and reasoning, critical thinking, problem solving, reading comprehension, and logical reasoning. | BMAT, TMUA, LNAT, and TSA | 73.0 |
| Oh et al [11] | ChatGPT | The data set comprised 280 questions from the Korean General Surgery Board examinations conducted between 2020 and 2022. | Korean General Surgery Board examinations conducted between 2020 and 2022 | 76.4 |
| Angel et al [12] | GPT-4, GPT-3, Bard | "Anesthesia Review: 1000 Questions and Answers to Blast the BASICS and Ace the ADVANCED": multiple choice questions and possible answers from both basic and advanced sample examinations were entered into GPT-3 and GPT-4 using the ChatGPT plus user interface20 and entered into Bard using the user interface provided by Google.21 The questions were entered individually, and the answers from the AI^e^ were recorded. | American Board of Anesthesiology (ABA) Exam | GPT-4: 80.0  GPT-3: 50.0  Bard: 46.0 |
| Huang et al [13] | ChatGPT-4, ChatGPT-3 | The Radiation Oncology in-Training (TXIT) examination contains 300 questions covering various topics of radiation oncology. The 2022 Gray Zone collection contains 15 complex clinical cases. | 38th American College of Radiology (ACR) TXIT examination | - ChatGPT-4: 78.8 - ChatGPT-3: 62.1 |
| Oztermeli et al [14] | ChatGPT-3.5 | Publicly available medical specialty examination (MSE) questions and answer keys from the past 5 years were scanned, and a total of 1177 questions were included in the study. All questions were asked to ChatGPT, GPT-3.5 series, which is the March 23, 2023, version. The average score and rank that ChatGPT would receive if it had entered the examination that year were determined. Questions were categorized into short-question group, long-question group, single-select multiple-choice questions, and multiselect multiple-choice questions. | 5 MSEs | 70.9 |
| Gencer et al [15] | ChatGPT | ChatGPT was provided with a total of 105 questions divided into 7 distinct groups, each of which contained 15 questions. | Turkish language thoracic surgery examination | 90.5 |
| Guerra et al [16] | ChatGPT | GPT-4's performance was examined on 643 Congress of Neurological Surgeons Self-Assessment Neurosurgery (SANS) exam board-style questions from various neurosurgery subspecialties. Of these, 477 (74.2%) were text-based questions and 166 (25.8%) contained images. GPT-4 refused to answer 52 (8.1%) questions that contained no text. The remaining 591 (91.9%) questions were inputted into GPT-4, and its performance was evaluated based on first-time responses. Raw scores were analyzed across subspecialties and question types and then compared to previous findings on ChatGPT performance against SANS users, medical students, and neurosurgery residents. | Neurosurgery board examinations | 76.6 |
| Wang et al [17] | ChatGPT | Chinese National Medical Licensing Examination (CNMLE) in 2020, 2021, and 2022. Each set of questions consists of 4 units, with 150 questions per unit. Based on the requirements to pass the NMLE, a total score of 360 or above is considered qualified. | CNMLE | 47.0 |
| Alessandri Bonetti et al [18] | ChatGPT | GPT-3 was used in June 2023 to undertake the 2022 IRANE (Italian Residency Admission National Exam), a computer-based examination with 140 multiple-choice questions, taken by all Italian medical graduates yearly, used to assess basic science and applied medical knowledge. The examination was scored using the same criteria defined by the national educational governing body. The performance of ChatGPT was compared to the performance of the 15,869 medical graduates who took the examination in July 2022. Lastly, the integrity and quality of ChatGPT's responses were evaluated. | Italian Residency Admission National Exam | 87.1 |
| Weng et al [19] | ChatGPT | Taiwan's 2022 Family Medicine Board Exam, which combined both Chinese and English and covered various question types, including reverse questions and multiple-choice questions, and mainly focused on general medical knowledge. Each question was pasted into ChatGPT and its response recorded and compared with the correct answer provided by the examination board. SAS 9.4 and Microsoft Excel were used to calculate the accuracy rates for each question type. | Taiwan's 2022 Family Medicine Board Exam | 41.6 |
| Huang et al [20] | ChatGPT | The AI chatbot's responses were manually reviewed to determine the selected answer, response length, response time, provision of a rationale for the outputted response, and the root cause of all incorrect responses (classified into arithmetic, logical, and information errors). The performance of AI chatbots was compared against a cohort of family medicine residents who concurrently attempted the test. | University of Toronto Family Medicine Residency Progress Test (UTFMRPT) | 82.4 |
| Flores-Cohaila et al [21] | ChatGPT | Peruvian National Licensing Medical Examination (PNLME): ENAM 2022 data set, which consisted of 180 multiple-choice questions, to evaluate the performance of ChatGPT. Various prompts were used, and accuracy was evaluated. The performance of ChatGPT was compared to that of a sample of 1025 examinees. Factors such as question type, Peruvian-specific knowledge, discrimination, difficulty, quality of questions, and subject were analyzed to determine their influence on incorrect answers. Questions that received incorrect answers underwent a 3-step process involving different prompts to explore the potential impact of adding roles and contexts on ChatGPT's accuracy. | Peruvian ENAM | 86.0 |
| Beaulieu-Jones et al [22] | GPT-4 | Evaluated the performance of ChatGPT-4 on 2 surgical knowledge assessments: the SCORE (Surgical Council on Resident Education) and a second commonly used knowledge assessment, referred to as Data-B. Questions were entered in 2 formats: open ended and multiple choice. ChatGPT output was assessed for accuracy and insights by surgeon evaluators. | SCORE, Data-B | - SCORE: 71.0 - Data-B: 68.0 |
| Kufel et al [23] | GPT-3.5 | Państwowy Egzamin Specjalizacyjny (PES) consisting of 120 questions, provided by the Medical Examinations Center in Lodz. Questions were administered using the openai.com platform that grants free access to the GPT-3.5 model. | Polish radiology examination (PRE) | 52.0 |
| Huynh et al [24] | ChatGPT | 135 questions from the 2022 Self-assessment Study Program for Urology, with 3 independent researchers and 2 physician adjudicators. | Self-assessment Study Program for Urology | 28.0 |
| Borchert et al [25] | ChatGPT | UK Foundation Programme Office (UKFPO) 2023 Situational Judgement Test (SJT) practice examination entered into ChatGPT, scored on the FPO template; questions categorized according to domains of Good Medical Practice. | UK SJT | 76.0 |
| Skalidid et al [26] | ChatGPT | European Exam in Core Cardiology (EECC), the final examination for the completion of specialty training in cardiology in many countries. | EECC | 59.0 |
| Mannam et al [27] | ChatGPT | Using the Congress of Neurological Surgeons (CNS) SANS exam board review prep questions, we conducted 3 rounds of analysis with ChatGPT. We developed a novel ChatGPT Neurosurgical Evaluation Matrix (CNEM) to assess the output quality, accuracy, concordance, and clarity of ChatGPT answers. | SANS | 67.0 |
| Bolton et al [28] | PubMedGPT | PubMedGPT 2.7B, a new language model trained exclusively on biomedical abstracts and papers. This GPT-style model can achieve strong results on a variety of biomedical natural language processing (NLP) tasks, including a new state-of-the-art performance of 50.3% accuracy on the MedQA biomedical question-answering task. | USMLE | 50.3 |
| Yasunaga et al [29] | BioLinkBERT | LinkBERT, a language model pretraining method that leverages links between documents (eg, hyperlinks). Given a text corpus, we viewed it as a graph of documents and create language model inputs by placing linked documents in the same context. We then pretrain the language model with 2 joint self-supervised objectives: masked language modeling and our new proposal, document relation prediction. | USMLE | 45.1 |
| Gu et al [30] | PubMedBERT | Domain-specific pretraining can benefit by starting from general-domain language models. In this paper, we challenged this assumption by showing that for domains with abundant unlabeled text, such as biomedicine, pretraining language models from scratch results in substantial gains over continual pretraining of general-domain language models. | USMLE | 38.1 |
| Taylor et al [31] | Galactica | Large scientific corpus of papers, reference material, knowledge bases and many other sources. | USMLE | 44.4 |
| Yasunaga et al [32] | DRAGON^f^ | DRAGON, a self-supervised approach to pretraining a deeply joint language knowledge foundation model from text and knowledge graphs (KGs) at scale. | USMLE | 47.5 |

^a^LLM: large language model.

^b^Med-PaLM 2: Medical Patient Language Model 2.

^c^USMLE: United States Medical Licensing Examination.

^d^GPT: Generative Pretrained Transformer.

^e^AI: artificial intelligence.

^f^DRAGON: Deep Bidirectional Language-Knowledge Graph Pretraining.

## References

1. Singhal K, Tu T, Gottweis J, Sayres R, Wulczyn E, Hou L, Clark K, Pfohl S, Cole-Lewis H, Neal D, Schaekermann M, Wang A, Amin M, Lachgar S, Mansfield P, Prakash S, Green B, Dominowska E, Arcas B, Natarajan V. (2023). Towards expert-level medical question answering with large language models. https://cloud.google.com/blog/topics/healthcare-life-sciences/sharing-google-med-palm-2-medical-large-language-model
2. Venigalla A, Frankle J, Carbin M. BioMedLM: a domain-specific large language model for biomedical text. The Stanford Center for Research on Foundation Models (CRFM) and MosaicML. Dec. 2022. https://www.mosaicml.com/blog/introducing-pubmed-gpt
3. Sharma P, Thapa K, Dhakal P, Upadhaya MD, Adhikari S, Khanal SR. 2023. Performance of ChatGPT on USMLE: unlocking the potential of large language models for AI-assisted medical education. *arXiv preprint arXiv:2307.00112*.
4. Kung TH, Cheatham M, Medenilla A, Sillos C, De Leon L, Elepaño C, Madriaga M, Aggabao R, Diaz-Candido G, Maningo J, Tseng V. Performance of ChatGPT on USMLE: potential for AI-assisted medical education using large language models. *PLOS Digit Health*. 2023 Feb 9;2(2):e0000198. doi: 10.1371/journal.pdig.0000198. PMID: 36812645; PMCID: PMC9931230.
5. Raimondi R, Tzoumas N, Salisbury T, et al. Comparative analysis of large language models in the Royal College of Ophthalmologists fellowship exams. *Eye (Lond)*. 2023 Dec; 37(17):3530-3533. https://doi.org/10.1038/s41433-023-02563-3.
6. Gilson A, Safranek CW, Huang T, Socrates V, Chi L, Taylor RA, Chartash D. How does ChatGPT perform on the United States Medical Licensing Examination? The implications of large language models for medical education and knowledge assessment. *JMIR Med Educ*. 2023 Feb 8;9:e45312. doi: 10.2196/45312. PMID: 36753318; PMCID: PMC9947764.
7. Strong E, DiGiammarino A, Weng Y, Basaviah P, Hosamani P, Kumar A, Nevins A, Kugler J, Hom J, Chen JH. Performance of ChatGPT on free-response, clinical reasoning exams. *medRxiv [Preprint]*. 2023 Mar 29:2023.03.24.23287731. doi: 10.1101/2023.03.24.23287731. PMID: 37034742; PMCID: PMC10081420.
8. Morreel S, Mathysen D, Verhoeven V. Aye, AI! ChatGPT passes multiple-choice family medicine exam. *Med Teach*. 2023 Mar 11:1. doi: 10.1080/0142159X.2023.2187684. Epub ahead of print. PMID: 36905610.
9. Humar P, Asaad M, Bengur FB, Nguyen V. ChatGPT is equivalent to first year plastic surgery residents: evaluation of ChatGPT on the plastic surgery in-service exam. *Aesthet Surg J*. 2023 May 4:sjad130. doi: 10.1093/asj/sjad130. Epub ahead of print. PMID: 37140001.
10. Giannos P, Delardas O. Performance of ChatGPT on UK standardized admission tests: insights from the BMAT, TMUA, LNAT, and TSA examinations. *JMIR Med Educ*. 2023 Apr 26;9:e47737. doi: 10.2196/47737. PMID: 37099373.
11. Oh N, Choi GS, Lee WY. ChatGPT goes to the operating room: evaluating GPT-4 performance and its potential in surgical education and training in the era of large language models. *Ann Surg Treat Res*. 2023;104(5):269-273. doi:10.4174/astr.2023.104.5.269.
12. Angel MC, Rinehart JB, Canneson MP, Baldi P. Clinical knowledge and reasoning abilities of AI large language models in anesthesiology: a comparative study on the ABA exam. *medRxiv [Preprint]*. 2023 May 16:2023.05.10.23289805. doi: 10.1101/2023.05.10.23289805. PMID: 37292642; PMCID: PMC10246030.
13. Huang Y, Gomaa A, Semrau S, Haderlein M, Lettmaier S, Weissmann T, Grigo J, Tkhayat HB, Frey B, Gaipl U, Distel L, Maier A, Fietkau R, Bert C, Putz F. Benchmarking ChatGPT-4 on a radiation oncology in-training exam and Red Journal Gray Zone cases: potentials and challenges for ai-assisted medical education and decision making in radiation oncology. *Front Oncol*. 2023 Sep 14;13:1265024. doi: 10.3389/fonc.2023.1265024. PMID: 37790756; PMCID: PMC10543650.
14. Oztermeli AD, Oztermeli A. ChatGPT performance in the medical specialty exam: an observational study. *Medicine (Baltimore)*. 2023 Aug 11;102(32):e34673. doi: 10.1097/MD.0000000000034673. PMID: 37565917; PMCID: PMC10419419.
15. Gencer A, Aydin S. Can ChatGPT pass the thoracic surgery exam? *Am J Med Sci*. 2023 Oct;366(4):291-295. doi: 10.1016/j.amjms.2023.08.001. Epub 2023 Aug 6. PMID: 37549788.
16. Guerra GA, Hofmann H, Sobhani S, Hofmann G, Gomez D, Soroudi D, Hopkins BS, Dallas J, Pangal DJ, Cheok S, Nguyen VN, Mack WJ, Zada G. GPT-4 artificial intelligence model outperforms ChatGPT, medical students, and neurosurgery residents on neurosurgery written board-like questions. *World Neurosurg*. 2023 Aug 18:S1878-8750(23)01144-0. doi: 10.1016/j.wneu.2023.08.042. Epub ahead of print. PMID: 37597659.
17. Wang X, Gong Z, Wang G, Jia J, Xu Y, Zhao J, Fan Q, Wu S, Hu W, Li X. ChatGPT performs on the Chinese National Medical Licensing Examination. *J Med Syst*. 2023 Aug 15;47(1):86. doi: 10.1007/s10916-023-01961-0. PMID: 37581690.
18. Alessandri Bonetti M, Giorgino R, Gallo Afflitto G, De Lorenzi F, Egro FM. How does ChatGPT perform on the Italian Residency Admission National Exam compared to 15,869 medical graduates? *Ann Biomed Eng*. 2023 Jul 25. doi: 10.1007/s10439-023-03318-7. Epub ahead of print. PMID: 37490183.
19. Weng TL, Wang YM, Chang S, Chen TJ, Hwang SJ. ChatGPT failed Taiwan's Family Medicine Board Exam. *J Chin Med Assoc*. 2023 Aug 1;86(8):762-766. doi: 10.1097/JCMA.0000000000000946. Epub 2023 Jun 9. PMID: 37294147.
20. Huang RS, Lu KJQ, Meaney C, Kemppainen J, Punnett A, Leung FH. Assessment of resident and AI chatbot performance on the University of Toronto Family Medicine Residency Progress Test: comparative study. *JMIR Med Educ*. 2023 Sep 19;9:e50514. doi: 10.2196/50514. PMID: 37725411; PMCID: PMC10548315.
21. Flores-Cohaila JA, García-Vicente A, Vizcarra-Jiménez SF, De la Cruz-Galán JP, Gutiérrez-Arratia JD, Quiroga Torres BG, Taype-Rondan A. Performance of ChatGPT on the Peruvian National Licensing Medical Examination: cross-sectional study. *JMIR Med Educ*. 2023 Sep 28;9:e48039. doi: 10.2196/48039. PMID: 37768724.
22. Beaulieu-Jones BR, Shah S, Berrigan MT, Marwaha JS, Lai SL, Brat GA. Evaluating capabilities of large language models: performance of GPT4 on surgical knowledge assessments. *medRxiv [Preprint]*. 2023 Jul 24:2023.07.16.23292743. doi: 10.1101/2023.07.16.23292743. PMID: 37502981; PMCID: PMC10371188.
23. Kufel, J., Paszkiewicz, I., Bielówka, M., Bartnikowska, W., Janik, M., Stencel, M., Czogalik, Ł., Gruszczyńska, K., & Mielcarska, S. (2023). Will ChatGPT pass the Polish specialty exam in radiology and diagnostic imaging? Insights into strengths and limitations. *Polish J Radiol*, 88, e430–e434. https://doi.org/10.5114/pjr.2023.131215
24. Huynh LM, Bonebrake BT, Schultis K, Quach A, Deibert CM. New artificial intelligence ChatGPT performa poorly on the 2022 Self-assessment Study Program for urology. *Urol Pract*. 2023 Jul;10(4):409-415. doi: 10.1097/UPJ.0000000000000406.Epub 2023 Jun 5. PMID 37276372.
25. Borchert RJ, Hickman CR, Pepys J, Sadler TJ. Performance of ChatGPT on the Situational Judgement Test – a professional dilemma-based examination for doctors in the United Kingdom. *JMIR Med Educ*. 2023 Aug 7;9:e48978. Doi:10.2196/48978. PMID:37548997; PMCID: PMC10442724.
26. Skalidid I, Cagnina A, Luangphiphat W, Mahendiran T, Muller O, Abbe E, Fournier S. ChatGPT takes on the European Exam in Core Cardiology: an artificial intelligence success story? *Eur Heart J Digit Health*. 2023 Apr 24;4(3):279-281. doi: 10.1093/ehjdh/ztad029.
27. Mannam SS, Subtirelu R, Chauhan D, Ahmad H, Matache IM, Bryan K, Chitta SVK, Bathula SC, Turlip R, Wathen C, Ghenbot Y, Ajmera S, Blue R, Chen I, Ali Z, Malhotra N, Srinivasan V, Ozturk A, Yoon JW. (2023). Large language model-based neurosurgical evaluation matrix: a novel scoring criteria to assess the efficacy of ChatGPT as an educational tool for neurosurgery board preparation. *World Neurosurg*, S1878-8750(23)01448-1. Advance online publication. https://doi.org/10.1016/j.wneu.2023.10.043.
28. Bolton E, Hall D, Yasunaga M, Lee T, Manning C, Liang P. Stanford CRFM introduces PubMedGPT 2.7B. https://hai.stanford.edu/news/stanford-crfm-introduces-pubmedgpt-27b. 2022.
29. Yasunaga M, Leskovec J, Liang P. LinkBERT: pretraining language models with document links. *arXiv preprint arXiv:2203.15827* (2022).
30. Gu Y, Tinn R, Cheng H, Lucas M, Usuyama N, Liu X, Naumann T, Gao J, Poon H. Domain-specific language model pretraining for biomedical natural language processing. *ACM Trans Comput Healthc (HEALTH)* 3:1-23 (2021).
31. Taylor R, Kardas M, Cucurull G, Scialom T, Hartshorn A, Saravia E, Poulton A, Kerkez V, Stojnic R. Galactica: a large language model for science. *arXiv preprint arXiv:2211.09085* (2022).
32. Yasunaga M, Bosselut A, Ren H, Zhang X, Manning CD, Liang PS, Leskovec J. (2022). Deep bidirectional language-knowledge graph pretraining. *Adv Neural Inf Proc Syst*, 35:37309-37323.
