# Supplementary figures and images for: The Accuracy and Capability of Artificial Intelligence Solutions in Health Care Examinations and Certificates: Systematic Review and Meta-Analysis
Source: J Med Internet Res. 2024 Nov 5;26:e56532. doi: 10.2196/56532 (PMC11576595; doi:10.2196/56532)

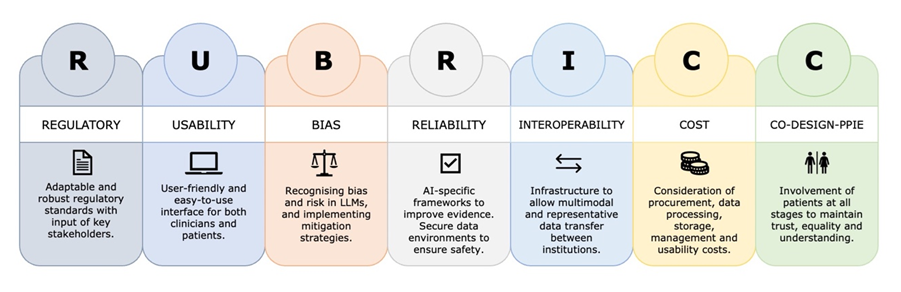

Supplement: Multimedia Appendix 2 [file jmir_v26i1e56532_app2.png]
